# A mixed-methods dataset on maternal reported experiences of breastfeeding difficulties and mental health in the UK

**DOI:** 10.1016/j.dib.2025.111700

**Published:** 2025-05-27

**Authors:** Elizabeth Braithwaite, Nicky Wright, Joseph Keenan, Hannah Fawcett, Athina Kidd, Karen Hughes, Rebecca Pearson

**Affiliations:** a School of Psychology, Faculty of Health and Education, Manchester Metropolitan University, Manchester, UK; b School of Nursing and Public Health, Faculty of Health and Education, Manchester Metropolitan University, Manchester, UK

**Keywords:** Infant feeding, Breastfeeding challenges, Postnatal mental health, Depression, Anxiety

## Abstract

Here we provide information on data from an online survey, conducted in October 2023, of 2010 UK-based participants who gave birth to their first child within the past decade. Participants must have given their first-born child breastmilk at any timepoint, which could have been directly from the breast or expressed milk. The survey included questions on demographic characteristics, experiences of breastfeeding and any breastfeeding difficulties, the impact of breastfeeding difficulties on lifestyle and mental health, as well as information on perinatal and current depression and anxiety symptoms. The data set includes both qualitative and quantitative responses. Current and in-depth information on links between breastfeeding difficulties and maternal mental health are needed to formulate recommendations to policy makers and support groups. Additionally, this information will be useful for identifying prevention/intervention targets to both support the maintenance of breastfeeding and maternal mental health in the postnatal period.

Specifications Table

**Table:** 

Subject	Health Sciences, Medical Sciences & Pharmacology
Specific subject area	Perinatal mental health
Type of data	Raw, Processed – imputed missing data and summary scores calculated
Data collection	Quantitative and qualitative data were collected in cross-section from a sample of N=2010 participants in October 2023. Participants were presented with a survey which included questions on demographic characteristics of the participant and their first-born child, questions about their breastfeeding journey with their first-born child, questions about any experiences of breastfeeding difficulties, questions about pre-pregnancy, during-pregnancy and post-pregnancy experiences of depression and anxiety. Finally, participants completed two validated measures to assess current symptoms of anxiety (GAD-7) and depression (EPDS).
Data source location	Data were collected online in the United Kingdom*.*
Data accessibility	Repository name: Open Science Framework Data identification number: 10.17605/OSF.IO/3HWRX Direct URL to data: https://osf.io/3hwrx/
Related research article	

## 1 Value of the Data

• The relationship between breastfeeding challenges and maternal mental health is currently unclear and highly complex; there is evidence that women with poor mental health are less likely to breastfeed, but also that successful breastfeeding is associated with improvements in mental health. Little is known about the impact of breastfeeding difficulties on maternal mental health, and more contextual qualitative and quantitative research on this topic is needed to fully understand this complex relationship.
• Current and in-depth information on links between breastfeeding difficulties and maternal mental health are needed to formulate recommendations to policy makers and support groups.
• This data will be useful for identifying prevention/intervention targets to both support the maintenance of breastfeeding and maternal mental health in the postnatal period.

## 2 Background

Successful breastfeeding and good maternal mental health are globally recognised health goals and key to long-term child health. The World Health Organisation (WHO) recommend that infants are exclusively breastfed for the first 6 months of life [1], however the UK has one of the lowest breastfeeding rates in the world [2]. 81 % of mothers initiate breastfeeding, but just 1 % exclusively breastfeed to 6 months [3]. This may be because 70 % of mothers experience difficulties with breastfeeding [4]. Unsuccessful breastfeeding initiation [5], breastfeeding difficulties [5], and breastfeeding pain [6] have been associated with maternal depression. Additionally, breastfeeding support for mothers has changed drastically in the UK over the past decade with cuts to public health spending [7]. Now, parents have less access to support, but expectations to breastfeed are very high [8], following an intense period of public health campaigning to promote breastfeeding. Evidence regarding the specific impact of breastfeeding difficulties on maternal mental health is limited to small scale qualitative studies. This study was therefore designed to provide in-depth and up-to-date quantitative and qualitative data for scientists, clinicians, and policy makers to assist them in supporting both postnatal mental health and breastfeeding maintenance.

## 3 Data Description

Two anonymised datasets are available in both SAV and CSV formats on the Open Science Framework website [9], which can be imported into the most compatible statistical software packages. In the first dataset (labelled ‘Breastfeeding and mental health data – open access’), reverse coding was implemented where required for the current depression measure (Edinburgh Postnatal Depression Scale; EPDS) [10], but total scores for the depression and anxiety (General Anxiety Disorders-7; GAD7) scales have not been calculated. There is some missing data on the EPDS and GAD7 items, and any researchers wishing to use this data can address the missing data with respect to the total scores as they see fit. In the second dataset (labelled ‘Breastfeeding and mental health data – open access imputed data’) the missing data has been imputed and total scores created for the EPDS and GAD-7 measures. Details of the data imputation and total score creation is available in a PDF file labelled ‘Breastfeeding and Maternal Mental Health project data imputation and total scores’ and the syntax is available (‘Syntax1_Mental health and breastfeeding data imputation and total scores). In brief, 409 data points were missing across the 10 EPDS items and 200 data points were missing across the 7 GAD-7 items (0.7–3.8 % missing data). Data were imputed using the multiple imputation command on SPSS v29. A detailed data dictionary is also provided with the data as a PDF document labelled ‘Breastfeeding and Maternal Mental Health Data Dictionary’. The survey questionnaire and supporting documents (participant information sheet, consent form, and debrief) are also available to view and download as Microsoft Word documents [9].

Suitable software packages for viewing and analysing this data include Excel, SPSS, STATA, and R. The data dictionary provides detailed information about all the variables in the dataset and how they are coded. It also includes guidance on the best variables to use in analysis for child current age, birthweight, and gestational age at birth, and how to create the total scores for the EPDS and GAD-7 measures of current depression and anxiety symptoms from the raw data.

## 4 Experimental Design, Materials and Methods

**Design, sample, and procedure**. Quantitative and qualitative data were collected in cross-section from a sample of N=2010 participants in October 2023. UK-based participants were recruited from Prolific (www.prolific.com); a website provider which hosts online research studies. Prolific has over 12,000 participants signed up who wish to partake in online studies and are paid for their time. When Prolific was founded in 2014, participants were recruited via three channels: 1) social media, 2) flyer distribution at university campuses, and 3) the prolific referral scheme, which allowed participants to invite members of their social network to the platform in return for small cash incentives. Over the past decade, participants have continued to be recruited to Prolific via social media and word of mouth. The study was advertised to potential participants who reported (via Prolific’s screening questionnaires) that they had given birth to their first child within the last 10 years. Before the completion of the survey, potential participants were required to read an information sheet and provide informed consent. Here, participants confirmed that they 1) had read the participant information sheet, 2) had the opportunity to consider the information and ask questions, and had these answered satisfactorily, 3) understand that participation is voluntary and they are free to withdraw at any time by closing the browser, however once the survey is complete they will be unable to withdraw their data because it will be fully anonymised, 4) agreed to take part in the survey as described in the information sheet, 5) understand and agree that their words may be quoted anonymously in research outputs, and 6) give permission for a fully anonymised version of the dataset to be deposited in an open access repository (the Open Science Framework website, www.osf.io).

After participants consented to participate in the survey, screening questions were used to determine eligibility. The eligibility criteria for this study were: 1) the participant had given birth to their first child in past 10 years, 2) the first child was not one of a multiples birth (i.e. twins or triplets), 3) the first child was not born before 37 weeks gestation, 4) the participant gave their child breastmilk at any point (which included giving baby breastmilk directly from the breast and/or expressed milk), and 5) participant is aged 18 or over. The rationale for excluding participants who had a multiples birth or child born before 37 weeks gestation was because of the challenges associated with establishing breastfeeding under these circumstances. All questions in the survey concerned the participant’s experiences of breastfeeding their first child.

Following the screening questions, participants were presented with the survey, which included questions on demographic characteristics of the participant and their first-born child, questions about their breastfeeding journey with their first-born child, questions about any experiences of breastfeeding difficulties, questions about pre-pregnancy, during-pregnancy and post-pregnancy experiences of depression and anxiety. Finally, participants completed two validated measures to assess current symptoms of anxiety (GAD-7) and depression (EPDS). Participants were able to skip any questions that they did not want to answer. Following completion of the survey, participants were presented with a debrief which included contact information for mental health and breastfeeding support services for further support if required. Participants completed the survey anonymously. The survey used in this study, all supporting documents, data, and a data dictionary are freely available to view and download [9].

**Measures**. *Demographic characteristics of parent, child, and birth outcomes*. Participants reported their *age, ethnicity* (Asian or Asian British, Black/African/Caribbean/Black British, Mixed/Multiple Ethnic Groups, White, Other Ethnic Group (please describe)), and their highest *educational qualification* (Diploma, BTEC, GCSE, A Level, Undergraduate Degree, Postgraduate degree). They then reported the following information about their first-born child: *current age* (in years and months), *sex assigned at birth* (male, female, intersex, not sure, prefer not to say), *birthweight* (in kgs or lbs and oz), *gestational age at birth* (in weeks and days), *method of delivery* (vaginal, ventouse, forceps, planned c-section, emergency c-section), any *medical complications or illnesses* since birth (no, yes – please describe). A summary of the demographic characteristics of the sample is displayed in Table 1.

**Table 1:** Demographic characteristics of participants and their first-born child whom they reported on in this study.

Variable	N	%	Mean	SD	Range
Maternal Age	1991		34.59	5.104	20–53
Maternal Ethnicity	2010				
Asian or Asian British	86	4.3			
Black/African/Caribbean/Black British	61	3			
Mixed/Multiple Ethnic Groups	53	2.6			
White	1797	89.4			
Other	13	0.6			
Maternal Education	2005				
Diploma	91	4.5			
BTEC	60	3			
GCSE	150	7.5			
A Level	348	17.4			
Undergraduate Degree	868	43.2			
Postgraduate Degree	488	24.2			
Child's age in months	2001		61.70	30.97	1–118
Child's age in years	2013		5.14	2.581	0–9.8
Child's sex assigned at birth	2009				
Male	1035	51.5			
Female	969	48.3			
Not sure	1	0			
Prefer not to say	4	0.2			
Child's birthweight (grams)	1998		3406.14	502.22	907.18–5700
Child's gestational age at birth (days)	2007		278.98	9.54	259–392
Child's gestational age at birth (weeks)	2007		39.85	1.36	37–53.7
Child's method of delivery	2008				
Vaginal	977	48.7			
Ventouse	123	6.1			
Forceps	302	15			
Planned C-section	163	8.1			
Emergency C-section	443	22.1			

**Fig. 1:**
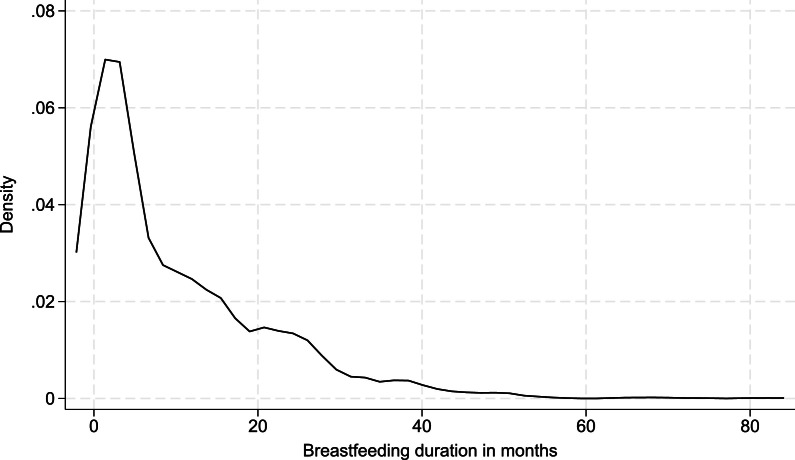
Kernel density plot showing the distribution of breastfeeding duration in months.

*Breastfeeding journey with first child*. Participants provided the following information about their breastfeeding journey with their firstborn child: *breastfeeding intentions* during pregnancy (yes, no, I wasn’t sure), *knowledge of how to breastfeed* prior to birth (no, yes – please describe). Participants were required to indicate *perceptions of pressure to breastfeed* during pregnancy and following birth from any of the following sources: partner, wider family member, friends, other parents of young children, midwife, health visitor, other healthcare professional, society, other – please describe. Participants indicated their response to these questions on a three-point Likert scale: 1=did not feel under any pressure at all to breastfeed, 2=somewhat felt pressured to breastfeed, 3=felt very pressured to breastfeed. Participants were asked to indicate *how long after birth they started breastfeeding* (in hours)*, how long they breastfed their first child* (in months), and whether they had received any *breastfeeding support* (no, yes – please describe). Fig.1 shows a density plot for the breastfeeding duration data. Participants were then asked to choose *three words to describe their experience of breastfeeding.* In this sample, 88 % of participants (n=1767) indicated that they intended to breastfeed whilst pregnant, but just 39.4 % (n=791) reported that they had any knowledge of how to breastfeed prior to birth, see Table 2. The mean time to breastfeeding initiation following birth was 3.49 h (SD=9.46, range=0–168 h), and on average participants breastfed their child for 9.96 months (SD=11.08, range=0–82). Note that we asked mothers about total breastfeeding duration, and not the duration of exclusive breastfeeding. We adopted a broad definition of breastfeeding, and asked participants to report on breastfeeding which included both feeding directly from the breast and feeding expressed milk. 52.5 % of participants (n=1054) reported that they received any breastfeeding support after birth.

**Table 2:** Participant-reported rates of breastfeeding intentions and knowledge in pregnancy, time to breastfeeding initiation, breastfeeding duration, and access to breastfeeding support.

Variable	Yes		No		Wasn't sure	
	N	%	N	%	N	%
Intention to breastfeed during pregnancy	1767	88	36	1.8	205	10.2
Knowledge of breastfeeding during pregnancy	791	39.4	1217	60.6		
	**Mean**	**SD**	**Range**	**N**		
Time to breastfeeding initiation (in hours)	3.49	9.46	0–168	1456		
Breastfeeding duration (in months)	9.96	11.08	0–82	1597		
	**Yes**		**No**			
	N	%	No	%		
Accessed breastfeeding support	1054	52.5	955	47.5		

*Experience of breastfeeding difficulties*. Participants were asked to indicate whether they experienced any of the following *breastfeeding difficulties* via a checkbox: sore nipples and/or nipple thrush, difficulty with latching, baby with tongue tie, mastitis, breast/nipple pain while feeding, breast engorgement, low milk supply, very high milk supply, other – please describe, none of the above. 96.9 % (n=1948) of participants indicated that they had experienced any breastfeeding challenges; the number of participants who reported experiencing each breastfeeding challenge is displayed in Table 3. If participants selected ‘none of the above’ they skipped to the next section of questions on mental health related to breastfeeding difficulties. However, if participants indicated that they experienced any of these breastfeeding difficulties, they were presented with further questions about their experience of breastfeeding difficulties. Participants were asked to choose *three words to describe how the experience of breastfeeding difficulties made them feel*. They were then asked whether their experience of breastfeeding difficulties made them *stop breastfeeding, reduce breastfeeding, or question whether they should continue breastfeeding* (yes, no, not sure). 77.6 % of participants (n=1505) reported that breastfeeding challenges made them consider whether they should continue breastfeeding, 49.4 % (n=953) reported that breastfeeding challenges caused them to reduce breastfeeding, and 44.9 % (n=868) of participants indicated that breastfeeding challenges caused them to stop breastfeeding, see Table 3. Participants were asked to indicate whether they felt that *breastfeeding difficulties impacted their mental health* (no, yes – please describe). 60.4 % of participants (n=1172) reported that the experience of breastfeeding challenges did impact their mental health, as displayed in Table 3. They were then asked to indicate whether their *experience of breastfeeding difficulties impacted them* in any of the following ways, and were able to indicate their response on a sliding scale from 1 (didn’t impact me at all) to 10 (impacted me a lot): going out and socialising less with family and friends, fewer opportunities for making new mum friends, negative impact on relationship with partner, not sleeping well, feeling in pain, worry about the baby’s feeding, worry about the baby’s development, feeling guilty about the baby’s feeding/development, eating a poorer diet, feeling like not a good enough mother, negative impact on identity as a mother.

**Table 3:** Participant reported rates of breastfeeding challenges and impacts on breastfeeding continuation and mental health.

Breastfeeding difficulties	N	%
Sore nipples and/or nipple thrush	1295	64.4
Difficulty with latching	1295	64.4
Baby with tongue tie	385	19.2
Mastitis	424	21.1
Breast/nipple pain when feeding	1148	57.1
Breast engorgement	910	45.3
Low milk supply	643	32.5
Very high milk supply	301	15.0
Other	166	8.3


Variable	Yes		No		Not sure	
	N	%	N	%	N	%
Breastfeeding challenges caused participant to stop breastfeeding	868	44.9	1025	53	201	2.2
Breastfeeding challenges caused participant to reduce breastfeeding	953	49.3	960	49.6	22	1.1
Breastfeeding challenges caused participant to question whether they should continue breastfeeding	1505	77.6	401	20.7	33	1.7
Breastfeeding challenges impacted participants mental health	1172	60.4	769	39.6	-	-

*History of depression and anxiety, and perinatal depression and anxiety*. Participants were asked to indicate whether they experienced a mental health problem prior to pregnancy, during their pregnancy, or since the birth of their first child, and were able to choose from the following responses: none, depression – I suspected or identified with being depressed, depression – a doctor told me that I had depression and/or referred me for help, anxiety – I suspected or identified with being anxious, anxiety – a doctor told me I had anxiety and/or referred me for help.

*Current symptoms of depression and anxiety*. Participants self-reported current symptoms of depression over the past 7 days using the Edinburgh Postnatal Depression Scale (EPDS), a widely used scale to assess symptoms of depression in parents [10]. The scale consists of ten items that describe common symptoms of depression, e.g. ‘I have blamed myself unnecessarily when things went wrong’, and ‘I have felt scared or panicky for no good reason’. Each item is scored from 0 to 3, and the scale has a maximum of 30, with a score of 13 or above indicative of clinically significant depression (Cronbach’s alpha=.77). Participants self-reported symptoms of anxiety using The Generalised Anxiety Disorder Assessment (GAD-7) [11]. This is a 7-item scale that requires participants to respond to statements about symptoms of anxiety that they may have felt over the past two weeks on a 4-point scale from “Not at all” to “Nearly every day”. Examples of items include ‘Trouble relaxing’ and ‘Becoming easily annoyed or irritable’. A total anxiety score is created by summing all items to create a score from 0 to 21. Scores of 5, 10 and 15 are used as the cut-off points for mild, moderate, and severe anxiety respectively (Cronbach’s alpha=.92).

**Data pre-processing**. Qualtics (www.qualtrics.com), a tool for creating online surveys, was used to collect the data. Raw data (without IP addresses) were exported from Qualtrics as an SPSS Statistics Data Document (SPSS Version 19). Preprocessing was then performed to filter out any incomplete (n=294) or duplicate (n=40) responses, and data from any respondents who did not agree to all the consent items, or who did not meet the eligibility criteria. This resulted in complete data from N=2010 participants. Participants reported their child’s age in years and months, and a new variable was created to describe child’s age in months alone (child_age_months_final). Participants reported their child’s gestational age at birth in weeks and days, and a new variable was created to describe gestational age in days alone (child_gest_age_days_final). Similarly, participants were able to report their child’s birthweight in either kgs or lbs and oz. A new variable was created to describe child birthweight in grams (child_birthweight_g_final).

**Fig. 2:**
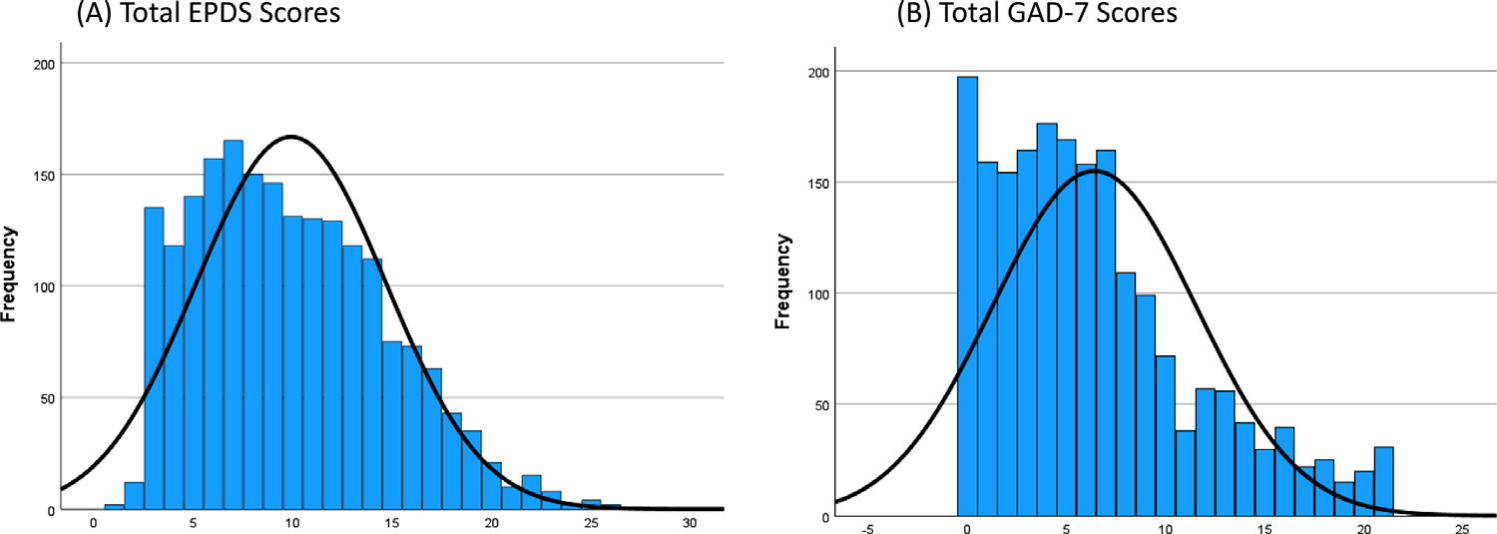
Histograms showing the distribution of total current depression (A) and anxiety (B) scores.

**Technical validation**. For the current purpose, the SAV file was exported from the Qualtrics online survey platform (Qualtrics, Provo, UT) where data were cleaned using SPSS v 29.0 (IBM Corp., Armonk, NT, USA). The dataset was carefully inspected for abnormal response patterns and completion times; neither were observed. Demographic characteristics of the sample were inspected. Reliability analysis was conducted for each of the psychometric measures included (EPDS for current symptoms of depression and GAD-7 for current symptoms of anxiety). The internal consistency (Cronbach’s alpha) of both scales was acceptable (EPDS α=0.77, GAD-7 α=0.92). Neither variable was skewed or kurtosed, see distribution of individual items in Fig. 2**.**

The mean score for the EPDS was 9.90 (SD=4.78, range=1–27, n=1997), and n=582 (29.14 %) scored above the cut-off of 12, which indicates clinically significant symptoms of depression [10]. This is higher than reports from the UK Office for National Statistics which estimated in 2022 that 19 % of women in the UK have depression [12], though they also highlight that over 1 in 3 women (35 %) aged 16 to 29 years experienced moderate to severe depressive symptoms [12]. 15 % of participants in the current study fall within this age range. There is also evidence that 35 % of UK mothers have a diagnosis of depression, with the majority being diagnosed during the child’s lifetime [13]. Self-reported symptoms of depression in this study are therefore in line with national reported averages of depression experienced by mothers. The mean GAD-7 score was 6.39 (SD=5.14, range=0-21, n=1997). In this sample of participants, n=153 (7.6 %) scored above the cut-off for severe anxiety, n=376 (18.7 %) scored above the cut off for moderate anxiety, and n=978 (48.7 %) scored above the cut off for mild anxiety. These rates are slightly lower than recent reports from the Office of National Statistics showing that in 2021/22, an average of 37.1 % of women reported high levels of anxiety [14]. A meta-analysis conducted in 2016 estimated that 8.5 % of mothers experience one or more anxiety disorders in the postpartum period [15], but data collected during the UK national lockdown because of the Covid-19 pandemic showed that 61 % of mothers self-reported clinically significant symptoms of anxiety [16]. The Royal College of Midwives have stated that an estimated half of cases of depression and anxiety in new mothers are undiagnosed [17], therefore in research studies such as this where mothers self-report symptoms of depression and anxiety, the reported rates of clinically-significant symptoms are likely to be higher than population estimates based on diagnoses.

Among UK women, those over the age of 30 and those who left education aged 18 or over are more likely to breastfeed [18], therefore our sample is somewhat representative of breastfeeding women in the UK.

## Limitations

The inclusion criteria for this study specified that participants must have given birth to their first child within the past 10 years, and participants reported on their experiences of breastfeeding with their first child. Thus, participants were retrospectively reporting on their experiences of breastfeeding and maternal mental health in the postnatal period, which may be subject to recall bias, social desirability bias, memory decay and positive or negative reframing. There is also evidence that accurate recall of breastfeeding experiences may vary by socio-demographic characteristics, such as education and smoking status [19]. Thus, researchers looking to use this data may wish to filter the data by age of the child, with the assumption that the younger the child is, the more accurate the recall of breastfeeding information by the participant. That said, there is evidence of good recall of breastfeeding experiences and information up to two decades postpartum [20]. Additionally, we recruited participants via Prolific, which is not representative of any national population: there is a female bias with a younger age and higher educational qualifications than the national average. Indeed, we find in our sample that the percentage of participants who identify as white and who hold University level qualifications is higher than the national average. Therefore, our sample may not be representative of all mothers in the UK.

## Ethics Statement

All participants provided informed consent before any data was collected, and all research procedures were carried out in accordance with the Declaration of Helsinki. Ethical approval for the study was granted by the Manchester Metropolitan University Faculty of Health and Education Research Ethics Committee (REF 58254).

## CRediT Author Statement

**Elizabeth Braithwaite**: Conceptualisation, data curation, formal analysis, funding acquisition, methodology, project administration, supervision, writing – original draft. **Nicky Wright**: Conceptualisation, methodology, writing – review & editing. **Joseph Keenan**: Conceptualisation, methodology, writing – review & editing. **Hannah Fawcett**: Methodology, writing – review & editing. **Athina Kidd**: Data curation, writing – review & editing. **Karen Hughes:** writing – review & editing. **Rebecca Pearson**: Conceptualisation, funding acquisition, methodology, writing – review & editing.

## Data Availability

Open Science FrameworkA mixed-methods dataset on maternal-reported experiences of breastfeeding difficulties and mental health (Original data)
